# Fibroblast Cell-Based Therapy for Experimental Autoimmune Diabetes

**DOI:** 10.1371/journal.pone.0146970

**Published:** 2016-01-14

**Authors:** Reza B. Jalili, Yun Zhang, Azadeh Hosseini-Tabatabaei, Ruhangiz T. Kilani, Mohsen Khosravi Maharlooei, Yunyuan Li, Sanam Salimi Elizei, Garth L. Warnock, Aziz Ghahary

**Affiliations:** Department of Surgery, University of British Columbia, Vancouver, BC, Canada; Children's Hospital Boston/Harvard Medical School, UNITED STATES

## Abstract

Type 1 diabetes (T1D) results from autoimmune destruction of insulin producing β cells of the pancreatic islets. Curbing autoimmunity at the initiation of T1D can result in recovery of residual β cells and consequently remission of diabetes. Here we report a cell-based therapy for autoimmune diabetes in non-obese diabetic (NOD) mice using dermal fibroblasts. This was achieved by a single injection of fibroblasts, expressing the immunoregulatory molecule indoleamine 2,3 dioxygenase (IDO), into peritoneal cavity of NOD mice shortly after the onset of overt hyperglycemia. Mice were then monitored for reversal of hyperglycemia and changes in inflammatory / regulatory T cell profiles. Blood glucose levels dropped into the normal range in 82% of NOD mice after receiving IDO-expressing fibroblasts while all control mice remained diabetic. We found significantly reduced islet inflammation, increased regulatory T cells, and decreased T helper 17 cells and β cell specific autoreactive CD8+ T cells following IDO cell therapy. We further showed that some of intraperitoneal injected fibroblasts migrated to local lymph nodes and expressed co-inhibitory molecules. These findings suggest that IDO fibroblasts therapy can reinstate self-tolerance and alleviate β cell autoreactivity in NOD mice, resulting in remission of autoimmune diabetes.

## Introduction

Type 1 diabetes (T1D) is an autoimmune disease that targets insulin producing β cells. The consequent loss of insulin production leads to elevated blood glucose, which in turn causes potentially lethal complications if left untreated [1]. Pathophysiology of T1D is complicated and several defects in immune regulation together with β cell inherent problems have been reported as contributing factors [1–3]. Numerous efforts have been made in confronting T1D from different aspects, unfortunately with limited success so far. Stem cells therapies has been in forefront of these interventions including application of bone marrow, embryonic, hepatic, pancreatic, adipose derived, and induced pluripotent stem cells (reviewed in [4&^5^]). Other cell types have also been used including lymphocytes conditioned by cord blood derived stem cells [6], autologous umbilical cord blood [7], or combined cell therapies [8]. Outcomes of these studies have led to the general conclusion that successful long-term reversal of T1D requires novel therapeutic strategies capable in tackling the disease from multiple fronts at the same time [9]. Such interventions should ideally be specific, effective, and long lasting with minimal adverse effects. Evidently, curing T1D requires reestablishment of immunological tolerance along with generation of new β-cells. To date, despite several promising combination therapies, no single treatment is known to be successful in accomplishing both of these aims simultaneously. Another problem in finding new therapies for T1D is that many immunotherapy strategies which were successful in non-obese diabetic (NOD) mice, have failed to show success in the clinical setting mainly because of fundamental differences between rodent and human T1D [10]. This necessitates development of new approaches to curb T1D more effectively.

Indoleamine 2,3 dioxygenase (IDO) is a rate-limiting enzyme in tryptophan catabolism with a potent tolerogenic capacity [11]. Defects in IDO pathway were correlated with autoimmune conditions including T1D [12]. Particularly, an impaired activity of IDO has been described in non-obese diabetic (NOD) mice as the experimental model for T1D [13,14]. As such, restoration of IDO function can be considered as a promising strategy for controlling autoimmunity in T1D. Indeed, IDO expressing dendritic [3,15,16] or Sertoli [17] cells have been used to achieve this goal. However, several limitations including difficulties associated with providing and maintaining sufficient quantities of these types of cells for large trials negatively impact their future clinical application.

Our group has previously used IDO-expressing dermal fibroblasts to suppress allograft rejection in skin and islet transplantation models [18,19]. Further, we showed that IDO-expressing fibroblasts are capable of converting naïve T cells into antigen specific regulatory T cells [20]. As such, here we investigated whether tolerogenic fibroblasts, generated by equipping these cells with IDO, can be employed as a potential tool for T1D immunotherapy.

## Material and Methods

### Experimental mice and intraperitoneal fibroblast injection

Female pre-diabetic non-obese diabetic (NOD) mice were purchased from the Jackson Laboratory (Bar Harbor, ME). Mice were kept in standard animal care facility until development of spontaneous diabetes. Dermal fibroblasts were explanted from mice skin and transduced with a lentiviral vector carrying IDO cDNA or a mock vector as described before [21]. IDO-expressing or control fibroblasts (2 × 10^7^ cells/ mouse) were injected in a single dose (400 μl) intraperitoneally (IP) to confirmed hyperglycemic NOD mice (blood glucose of 14–20 mmol/L) within a two-week frame following diabetes onset. These mice did not receive any other treatment. A subgroup of mice was also implanted with slow releasing 1-methyl tryptophan (1-MT) pellets to inhibit IDO enzymatic activity. Pellets were purchased from Innovative Research of America (Sarasota, FL) and were manufactured to deliver 5 mg/day of 1-MT over 14 days. After cell therapy, blood glucose levels were measured using an Accu-Chek Compact Plus blood glucose monitoring system twice a week. Mice were deemed diabetes-free when blood glucose levels decreased to <10 mmol/L. University of British Columbia (UBC) Animal Care Committee approved this study and all mice were cared for according to the guidelines of the UBC Animal Policy and Welfare Committee. Mice were kept in standard condition with all required welfare and full environmental enrichment. Mice were sacrificed at the endpoint of the study using inhalant anesthetic (isofluran), followed by carbon dioxide method according to UBC Animal Care Committee Policy.

### Histological analyses and immunostainings

Pancreases of mice were harvested at the endpoint of experiments (i.e. 6 weeks for the control group and 18 weeks for the IDO cell therapy group), fixed in 10% buffered formalin solution, and embedded in paraffin. Sections 5 μm thick from tissue were stained with hematoxylin and eosin and analyzed by light microscopy. Insulitis scoring was calculated according to the following criteria: severe insulitis, 50% or higher of the islet area is infiltrated; moderate insulitis, 50–25% of the islet area is infiltrated; mild or peri-insulitis, infiltration is less than 25% and restricted to the periphery of islets; and no insulitis, absence of cell infiltration. Insulin Immunofluorescence staining was performed using guinea pig anti-insulin antibody (1:500 dilution, Dako Laboratories, Mississauga, ON, Canada). Secondary antibody (1:2000 dilution, abcam, Cambridge, MA) used was FITC-goat anti-guinea pig IgG.

### Characterization of immune cells

Spleen and pancreatic lymph nodes were harvested at the endpoint of experiments (i.e. 6 weeks for the control group and 18 weeks for the IDO cell therapy group) and single cell suspensions were prepared. Cell suspensions were then incubated with fluorescent conjugated antibodies (eBioscience, San Diego, CA) specific for particular lymphocyte markers (i.e. CD4, CD25, IL-17, and FoxP3) according to manufacturer’s protocol. Fluorescence dot plots were created using a BD FACS Calibur flow and Accuri C6 cytometry machines (BD Biosciences Pharmingen, Mississauga, ON, Canada) and were used to determine the percentage of positive cells labeled with the corresponding antibodies. To detect autoreactive cytotoxic T cells, immune cells were stained with a NRP-V7 high-avidity peptide/MHC class I tetramer (manufactured by Dr. R. Tan group at University of British Columbia [22]) for 3 hours, then with APC-conjugated anti-CD8 and PerCP-conjugated anti CD4 and anti B220 (ebioscience) for 30 minutes, all on ice. CD8^+^ Tetramer positivity was then determined using a lymphocyte gate and exclusion of CD4^+^ and B220^+^ cells.

### Reverse transcriptase PCR

Total RNA was isolated from cultured fibroblasts using RNeasy kit (Qiagen, Maryland). cDNA was synthesized using SuperScript first-strand synthesis system for RT-PCR (Invitrogen, Carlsbad, CA) according to the manufacturer’s protocol. Polymerase chain reaction (PCR) was then performed using primers sequences (5’-3’) as follows. Mouse CD274 (PD-L1): forward ACTTGTACGTGGTGGAGTATGGCA, reverse TGGCTGGATCCACGGAAATTCTCT; mouse CD273 (PD-L2): forward: CGTGACAGCCCCTAAAGAAG, reverse: GATGACCAGGCAACGGTACT; mouse IL-1ra forward: GACCCTGCAAGATGCAAGCC, reverse: CAGGACGGTCAGCCTCTAGT. IDO-1 forward: TTCAGCAGCAGACTACAAGAATGGCACAC, reverse: TAGATGCTCTTGTTGGGTTACCTTAACC. Amplified PCR products were then separated by 1% agarose gel electrophoresis and visualized with SYBER Safe DNA gel staining (Invitrogen) under UV light.

### Tracking intraperitoneal injected fibroblasts

Fibroblasts were labeled using PKH26 red fluorescent cell membrane labeling kit (Sigma, St. Louis, MO) according to manufacturer’s instruction and injected to NOD mice intraperitoneally. Mice were euthanized at four time points with 14 days intervals (i.e. week 2 to week 8). Cells were retrieved from peritoneal cavity (by lavage), mesentery, mesenteric lymph nodes, and retro-peritoneal lymph nodes. Single ell suspensions were prepared by collagenase digestion (type I, 1 mg/ mL, Sigma) of tissues. Cells were stained for CCR7, CD90.2 and PD-L1 (eBioscience) and examined using flow cytometry. Further, presence of migratory fibroblasts in lymph nodes was examined using confocal microscopy. T do so, PKH26 labeled control or IDO expressing fibroblasts (2 × 10^7^ cells/ mouse) were injected intraperitoneally to recently diabetic NOD mice. After 2 weeks, mice were euthanized and mesenteric lymph nodes were harvested, freshly frozen and embedded in Cryomatrix (Thermo Scientific). Frozen sections (7 μm thick) from lymph nodes were permeabilized with 0.1% Triton-X-100 in PBS, stained with DAPI and visualized using a confocal fluorescence microscope (Axio Observer Z1 inverted confocal with spinning disk, Carl Zeiss, Jena, Germany). Images were analyzed using Zen software (Zeiss, Jena, Germany).

### In vitro NOD lymphocyte co-culture with IDO-expressing or control fibroblasts

Mesenteric lymph nodes from recently diabetic NOD mice were removed under sterile conditions and single-cell suspensions were obtained by pressing the spleens through a 40 μm nylon cell strainer (VWR International, LCC, BD Falcon) with a 3 ml syringe plunger (BD bioscience, BD syringe) and rinsed through with RPMI 1640 medium (Thermo Scientific) enriched with 10% fetal bovine serum (FBS) and 1% anti-mycotic antibiotic (GIBCO). Viable cells were counted by the trypan blue exclusion method and frequency of CD4^+^ CD25^+^ FoxP3^+^ cells were measured using flow cytometry as described above. We then cultured these lymphocytes in 24 well plates (10^6^ cells in 1 mL /well) either alone (mono-culture) or in co-culture with control or IDO-expressing fibroblasts. These fibroblasts were pre-cultured in the wells (2 × 10^5^ cells /well) before adding lymphocytes as described before [20]. After 7 days remaining in mono- or co-cultures, lymphocytes were harvested and frequency of CD4^+^ CD25^+^ FoxP3^+^ cells were measured using flow cytometry as described above.

### Statistical analysis

Data are reported as mean ± standard deviation of three or more independent set of experiments. The statistical differences of mean values among treated and control groups were tested with one-way ANOVA followed by Post hoc comparisons using Bonferroni correction. Kaplan-Meier survival analysis with log-rank (Mantel-Cox) test was done to compare rate of diabetes reversal among treatment groups. *P*-values less than 0.05 were considered statistically significant.

## Results

### Single injection of IDO expressing fibroblasts reversed diabetes in NOD mice

**Fig 1:**
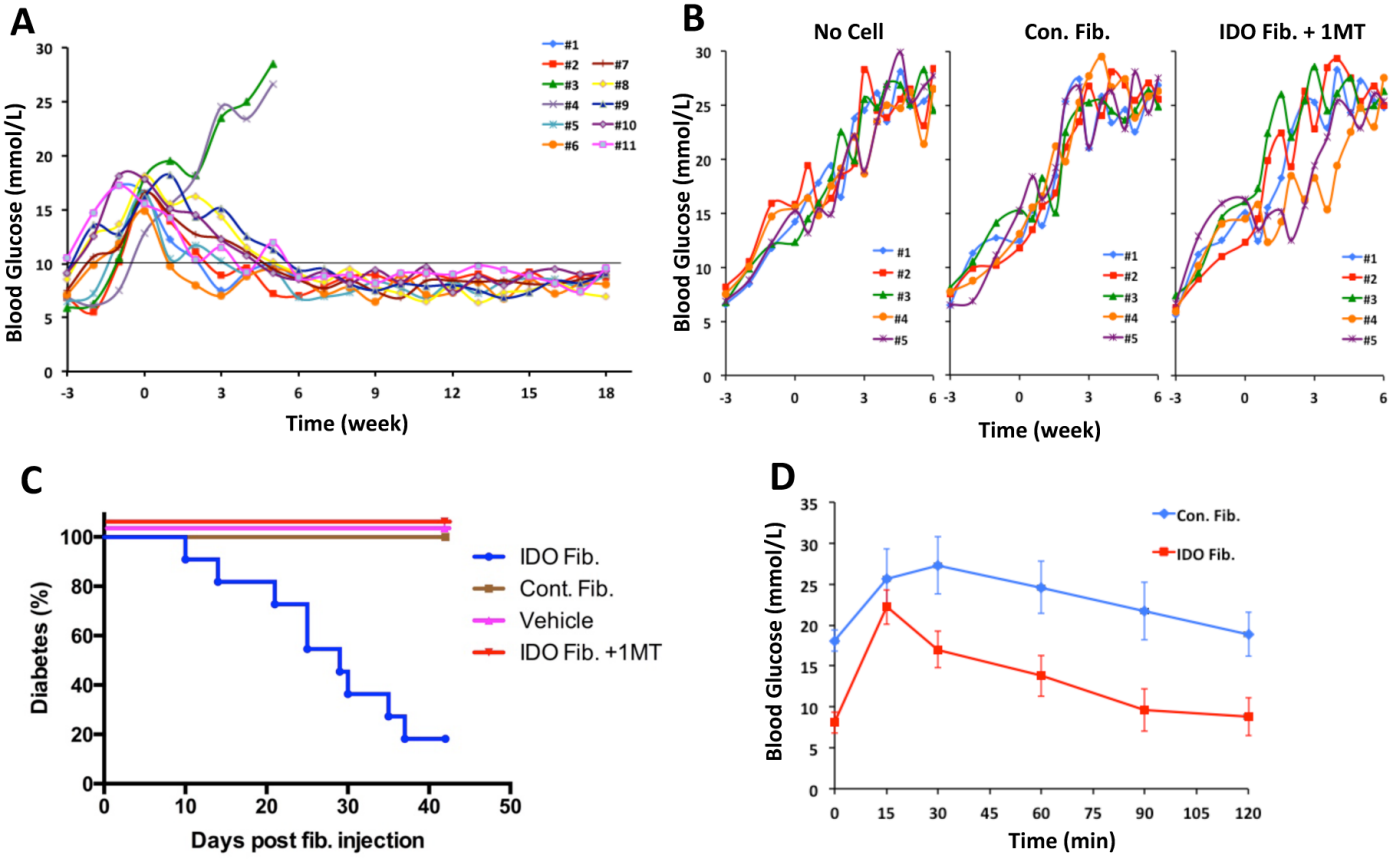
Reversal of hyperglycemia in NOD mice after cell therapy. IDO-expressing or control fibroblasts were injected intraperitoneally to NOD mice with overt hyperglycemia in the first two weeks following the onset of diabetes. These mice did not receive any other treatment. Blood glucose levels were checked twice a week. Panels **A** show blood glucose levels in IDO treated mice (*n* = 11 mice). Time point 0 denotes fibroblasts injection time. All control animals (**B**) that received no cells, control fibroblasts (Con. Fib.) or IDO expressing fibroblasts and an IDO inhibitor, 1-methyl tryptophan (IDO Fib. + 1MT) remained hyperglycemic (*n* = 5 mice/ group). Kaplan-Meier survival curve with log-rank analysis (**C**) showed significant decrease in diabetes rate of IDO fibroblast treated group. Intraperitoneal glucose tolerance tests (**D**) showed improvement of glycemic control in the IDO cell therapy group (red squares) compared to the control mice (blue diamonds).

Overtly diabetic NOD mice, within the first two weeks after hyperglycemia onset, received either a single intraperitoneal injection of IDO-expressing fibroblasts or control injections, including control fibroblasts (mock vector infected) or vehicle solution. To examine the role of IDO enzymatic function, a set of mice received intraperitoneal IDO fibroblasts in conjunction with an IDO inhibitor, 1-methyl tryptophan (1-MT), in the form of subcutaneous slow-releasing pellets. As shown in Fig 1A and 1B, while all control mice including those received 1-MT pellets sustained sever hyperglycemia, 9 out of 11 (82%) IDO cell therapy mice returned back to normoglycemic status. Successfully treated mice were kept for over 120 days (18 weeks). Kaplan-Meier survival analysis showed that IDO cell therapy significantly decreased diabetes frequency (P<0.0001, Fig 1C). Intraperitoneal glucose tolerance tests (IPGTT) conducted two weeks prior to endpoint of the study showed significantly improved glucose homeostasis in IDO cell therapy mice compared to control (diabetic) mice (Fig 1D) confirming the presence of a functional β cell mass in the IDO group. Overall, these data shows that a single IDO cell injection can very effectively reinstate normoglycemia in majority of severely diabetic NOD mice.

### IDO cell therapy decreased pancreatic insulitis and preserved insulin producing β cells

**Fig 2:**
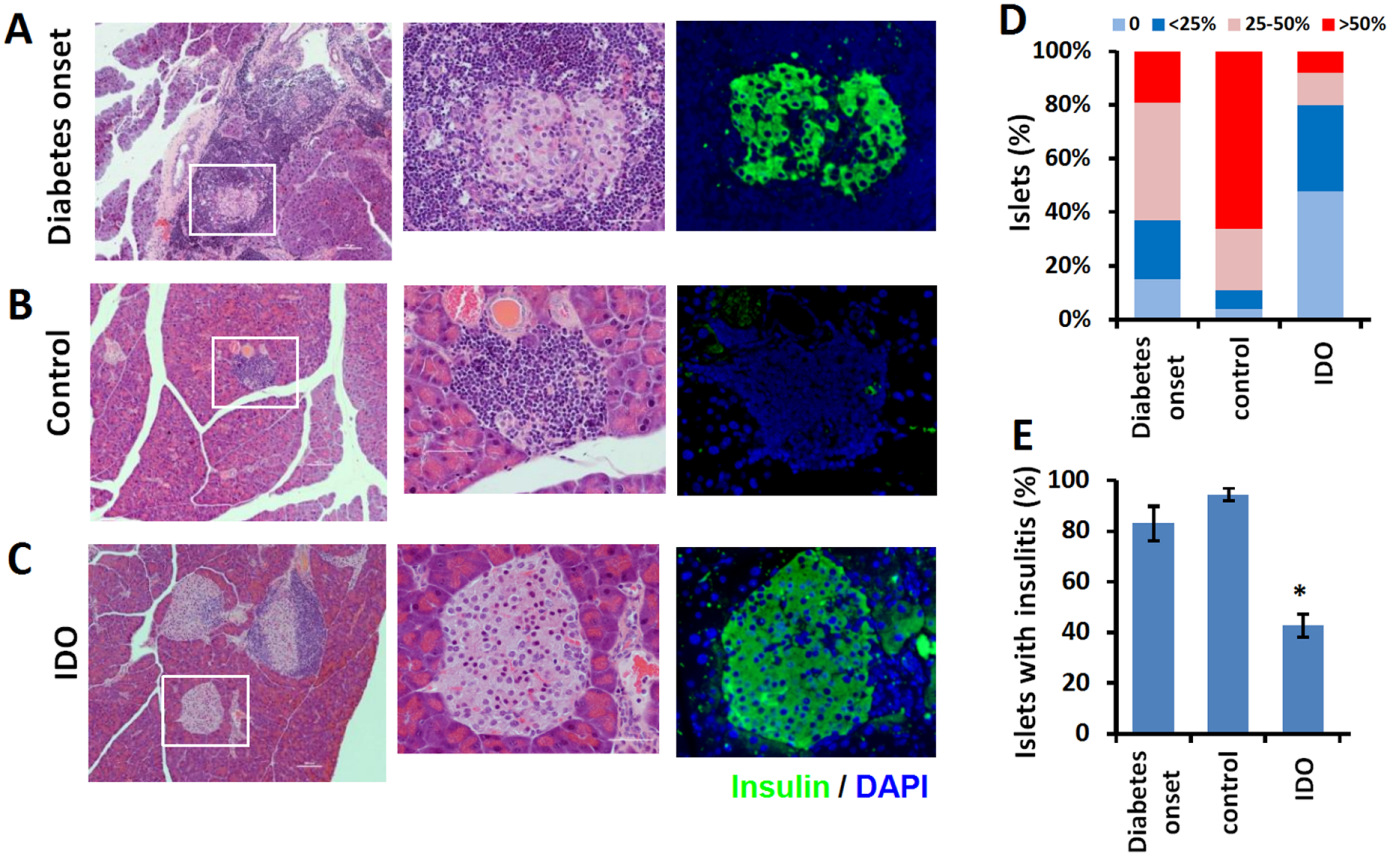
Histology of pancreas following cell therapy. Histology of representative pancreas sections of NOD mice before (at the commencement of autoimmune diabetes) and after cell therapy is shown. Panel **A** shows hematoxylin-eosin staining (left and center) and insulin immuno-staining (right) of pancreas sections of a NOD mouse euthanized in the first week after the onset of hyperglycemia. Panels **B** and **C** show pancreatic sections of an untreated control NOD mouse with chronic diabetes (6 weeks post-diabetes), an IDO cell treated NOD mouse (18 weeks post-treatment), respectively. Middle and right columns show higher magnification (×400) of the marked areas of the left column (×100). Panel **D** shows Insulitis scores calculated as described in Methods. The ratio (%) of islets with insulitis significantly decreased after IDO cell therapy animals compared to those of the early onset diabetic mice and controls (**E**). (*) denotes significant decrease in islet insulitis in IDO groups compared to the controls and diabetes onset (P<0.001, *n* = 5).

Pancreases of IDO treated and control mice were harvested at the endpoint of the study and stained with either H&E or immunostained for insulin. Pancreases of a group of NOD mice at the early stages of diabetes were also harvested and examined to demonstrate the condition of pancreas at the start point of the treatment. The results as presented in Fig 2 showed widespread insulitis (i.e. dense infiltrations of mononuclear cells into islets) and few remaining β cells at the beginning of the treatment (Fig 2A). In the control group, as expected, almost all β cells were destroyed after 6 weeks and islets were completely occupied with mononuclear cells (Fig 2B). In contrast, IDO cell therapy after 18 weeks resulted in significant clearance of insulitis and increasing in the number of insulin stained β cells (Fig 2C). Insulitis scores (Fig 2C), calculated as described in the Methods, showed significantly reduced inflammation in islets following IDO cell therapy with over 50% infiltration-free islets after 18 weeks (Fig 2E).

### Diabetes reversal by IDO cell therapy was associated with increased regulatory T cells

**Fig 3:**
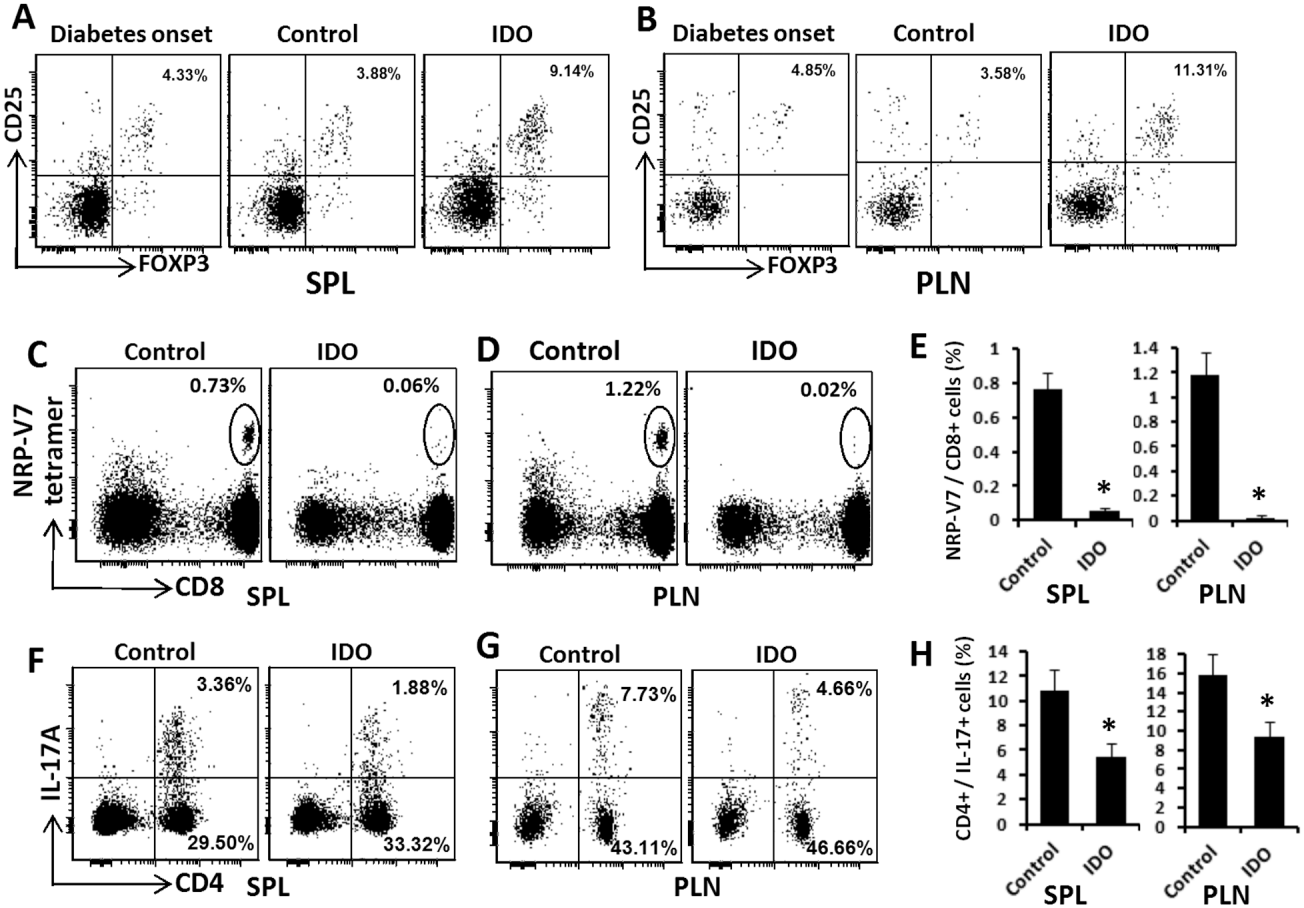
Frequency of regulatory, autoreactive CD8^+^, and Th_17_ T cells in NOD mice after cell therapy. NOD mice received IDO-expressing fibroblasts upon initiation of spontaneous diabetes. At the endpoint of the study, spleens and pancreatic lymph nodes of these mice were harvested and frequencies of various subtypes of T cells were measured using flow cytometry. Panels **A** & **B** show the frequencies of CD 25^+^ FoxP3^+^ regulatory T cells (gated on CD4^+^ T cells) in spleens (SPL) and pancreatic lymph nodes (PLN) of mice, respectively. Autoreactive T cells were detected using NRP-V7 high-avidity peptide/MHC class I tetramers as described in Methods. Panels **C** &**D** show representative plots for CD8^+^ tetramer positive cells in spleen (SPL) and pancreatic lymph nodes (PLN) using a lymphocyte gate and excluding CD4^+^ and B220^+^ cells. The numbers in upper right corner of plots indicate the percentage of CD8^+^ B220^-^ CD4^-^ tetramer^+^ cells. Panels **F** & **G** show representative plots for IL-17 and CD4 flow cytometry analysis of lymphocytes in spleens (SPL) and pancreatic lymph nodes (PLN). The numbers in upper right corner of plots indicate the percentage of CD4^+^ IL-17^+^ (Th_17_) cells. Panels **E** & **H** show quantification of the frequencies of autorective CD8^+^ T cells and Th_17_ cells, respectively. (*) denotes significant decrease of inflammatory cells in IDO groups compared to the controls (P<0.001, *n* = 5).

As noted earlier, IDO is a very potent inducer of regulatory T cell (Tregs). To investigate the emergence of a regulatory environment in response to IDO cell therapy, we measured the frequency of CD4^+^ CD25^+^ FOXP3^+^ Tregs in spleens and pancreatic lymph nodes of IDO treated and control mice. As shown in Fig 3, we found that IDO cell therapy significantly increased the frequency of splenic Tregs from 4.68% ± 1.01% at the onset of diabetes to 9.65% ± 1.04% (p< 0.001) following IDO cell therapy while frequency of Tregs in the control group showed no significant change (3.94% ± 1.00%, P> 0.05) compared to the diabetes onset. A similar increase in Tregs was also found in the pancreatic lymph nodes. While CD4^+^ CD25^+^ FOXP3^+^ were increased from 4.52% ± 1.03% to 11.82% ± 1.49% (P < 0.001), again no significant change was found in the control mice (3.83% ± 0.99%, P>0.05). Fig 3A & 3B show representative plots of Tregs in treated and control groups.

### IDO cell therapy decreased autoreactive and inflammatory immune cells

To gain a perspective on autoimmunity and inflammatory changes upon IDO cell therapy, we measured frequency of β cell specific autoreactive CD8^+^ T cells. To this end, we measured the frequency of NRP-V7 MHC I tetramer^+^ CD8^+^ (as described in Methods) and also Interleukin 17 (IL-17) producing CD4^+^ T cells (Th_17_ cells). The results as presented in the Fig 3C–3E show that CD8^+^ autoreactive T cells very significantly decreased and almost cleared in IDO cell therapy group compared to that of the control group both in the spleen (0.06% ± 0.01% vs. 0.76% ± 0.1%, P<0.001) and pancreatic lymph nodes (0.02% ± 0.01% vs. 1.18% ± 0.18%, P<0.001). Similarly, the frequency of pro-inflammatory Th_17_ cells showed a significant decrease in IDO group versus control group in both spleen (5.42% ± 1.16% vs. 10.84 ± 1.74%, P<0.001) and pancreatic lymph nodes (9.39% ± 1.58% vs. 15.76% ± 2.26%, P<0.001) (Fig 3F–3H).

### Intraperitoneal injected fibroblasts migrated to local lymph nodes and expressed co-inhibitory and anti-inflammatory molecules

**Fig 4:**
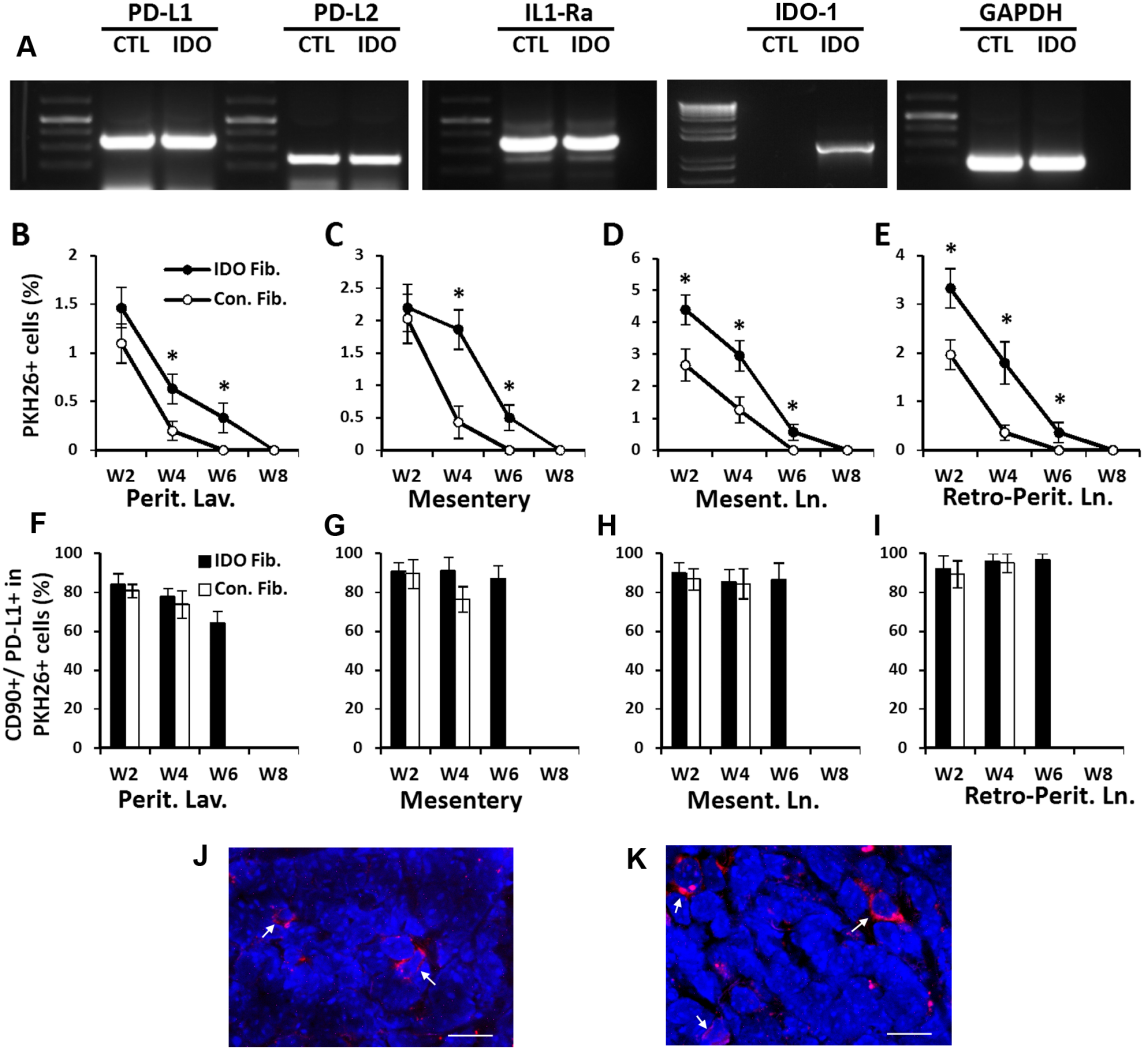
Expression of co-inhibitory and anti-inflammatory molecules by dermal fibroblasts and tracking of fibroblasts after intraperitoneal injection. IDO-expressing and control mouse dermal fibroblasts were examined for mRNA expression of co-inhibitory molecules Programmed Cell Death 1 and 2 ligands (PD-L1 and PD-L2), anti-inflammatory molecule interleukin-1 receptor antagonist (IL1-Ra), IDO-1, and GAPDH (as loading control) using reverse transcriptase PCR. Panel **A** shows high levels and almost equal expression of anti-inflammatory molecules in both control (CTL) and IDO-expressing fibroblasts. To track fibroblasts, IDO-expressing and control fibroblasts were labeled using PKH26 red fluorescent cell membrane labeling kit and injected to NOD mice intraperitoneally. Animals were euthanized at four time points with 14 days intervals (i.e. week 2 to week 8). Cells were harvested from peritoneal cavity (by lavage; Perit. Lav.), mesenteric membranous tissue (Mesentry), mesenteric lymph nodes (Mesent. Ln.), and retro-peritoneal lymph nodes (Retro-Perit. Ln.). Cells were stained for CD90.2 (a fibroblast marker) and PD-L1 and tested using flow cytometry. Panels **B** to **E** show frequency of PKH26+ cells at different time points (Week 2 to Week 8; W2to W8) in harvested tissues as indicated in the bottom of the panels. Black circles and white circles represent IDO-expressing and control fibroblasts, respectively. (*) denotes significant difference in frequency of PKH26^+^ in IDO fibroblast-injected mice versus control fibroblast-injected mice (p<0.001, *n* = 5). Panels **F** to **I** show frequencies of CD90^+^ PD-L1^+^ cells in PKH26^+^ gate. Black bars and white bars represent IDO-expressing and control fibroblasts, respectively. Panels J & K show frozen sections of mesenteric lymph nodes, two weeks following IP injection of control or IDO fibroblast, respectively. White arrows point to red fluorescent (PKH26^+^) cells among lymph node cells with blue colored nuclei (DAPI stained). Scale bar: 10 μm.

To scrutinize how fibroblast cell therapy affects the immune system, we tested expression of two important co-inhibitory cell surface markers i.e. programmed cell death ligand 1 and 2 (PD-L1, PD-L2) and also anti-inflammatory molecules, interleukin-1 receptor antagonist (IL-1RA). We fund that both primary and IDO-expressing fibroblasts produced these molecules (Fig 4A). It was also important to investigate the ultimate fate of the injected fibroblasts following intraperitoneal (IP) injection. To this end, we labeled IDO and control fibroblasts with PKH26 red fluorescent cell linker before IP injection. The presence and frequency of labeled fibroblasts were then examined in various tissues at different time points post-injection. The results showed that IP injected fibroblasts mainly remained in peritoneal cavity or attached to mesenteric membranes while some of them migrated to mesenteric and retroperitoneal lymph nodes (Fig 4B–4E). Examining cell surface markers confirmed that majority of those PKH26^+^ cells were CD90^+^ fibroblasts which also express PD-L1 (Fig 4F–4I). Interestingly, both control and IDO expressing fibroblasts showed similar phenotypic and functional characteristics after IP injection but the quantity of control fibroblasts that migrated was almost 2 times less than IDO fibroblasts and also control fibroblasts cleared faster (within 6 weeks) than IDO fibroblasts (within 8 weeks) (Fig 4B–4E).

To visualize the migratory fibroblasts within the lymph nodes, frozen sections from NOD mice mesenteric lymph nodes were examined 2 weeks after IP fibroblast injection using fluorescence confocal microscopy. As shown in Fig 4J and 4K, a few red PKH26 labeled cells were visible among lymphocytes. This finding was in line with our flow cytometric results showing 3–5% PKH26+ cells in lymph nodes (Fig 4D). We could not find any PKH26 labeled cells in other tissues including, blood circulation, spleen, distant lymph nodes (i.e. cervical), lung, liver, and pancreas (S1 Fig).

### IDO-expressing fibroblast increased frequency of regulatory T cell in vitro

**Fig 5:**
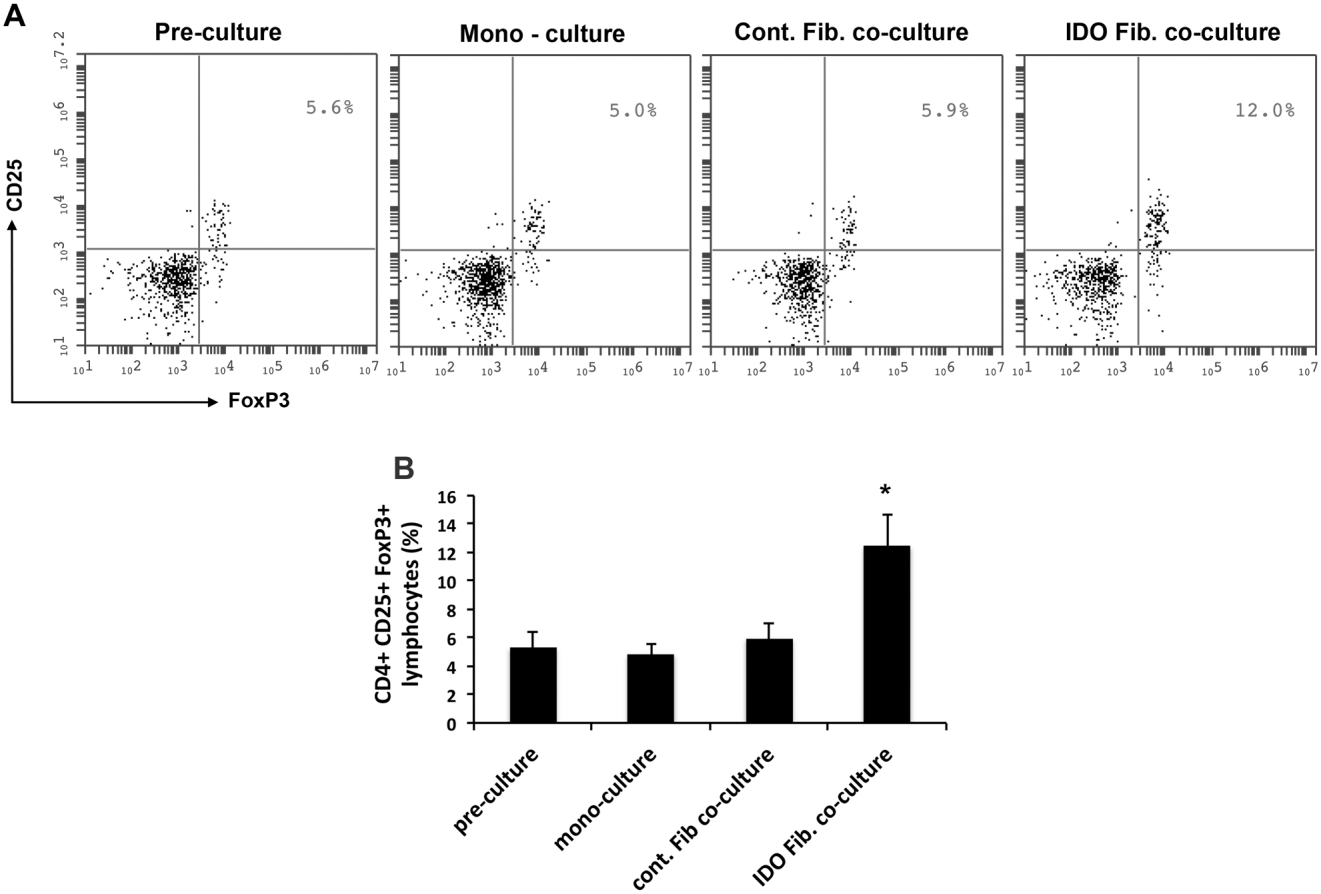
Increased frequency of CD4^+^ CD25^+^ FoxP3^+^ cells after co-culture with IDO-expressing fibroblast. Lymphocytes were isolated from mesenteric lymph nodes of recently diabetic NOD mice and were cultured either alone or co-cultured with control or IDO-expressing fibroblasts as described in Methods. The frequency of CD4^+^ gated CD25^+^ FoxP3^+^lypmocytes were measured using flow cytometry before culturing (Pre-culture) and after 7 days mono- or co-cultures. Panel A shows representative plots for CD25+ FoxP3+ regulatory T cells (gated on CD4+ T cells) and panel B shows frequencies of these cells in different experimental groups. (*) denotes statistically significant increase in the frequency of regulatory T cell after co-culturing with IDO-expressing fibroblasts compared to pre-culture amount (P<0.001, n = 3).

As noted above, we found that following IP injection; some fibroblasts migrated and homed in lymph nodes. To investigate the effect of these fibroblasts on immune cells, we isolated lymphocytes from recently diabetic NOD mice and co-cultured them with IDO-expressing or control fibroblast *in vitro* as described in Methods. As shown in Fig 5, we found that the frequency of CD4^+^ CD25^+^ FoxP3^+^ cells significantly increased from 5.3% ± 1.1% to 12.5% ± 2.3% (p<0.001) after 7 days co-culture with IDO-expressing fibroblasts. However, this frequency remained unchanged in mono-cultured lymphocytes (4.9% ± 0.71%) and those co-cultured with control fibroblast (5.84% ± 1.0%) when compared to pre-culture frequency (5.3% ± 1.1%, p>0.5). This finding suggests that IDO expressing fibroblast, after homing to lymph nodes, can increase the frequency of regulatory T cells as one possible mechanism for controlling autoimmunity.

### Migratory fibroblasts expressed CC-chemokine receptor 7

**Fig 6:**
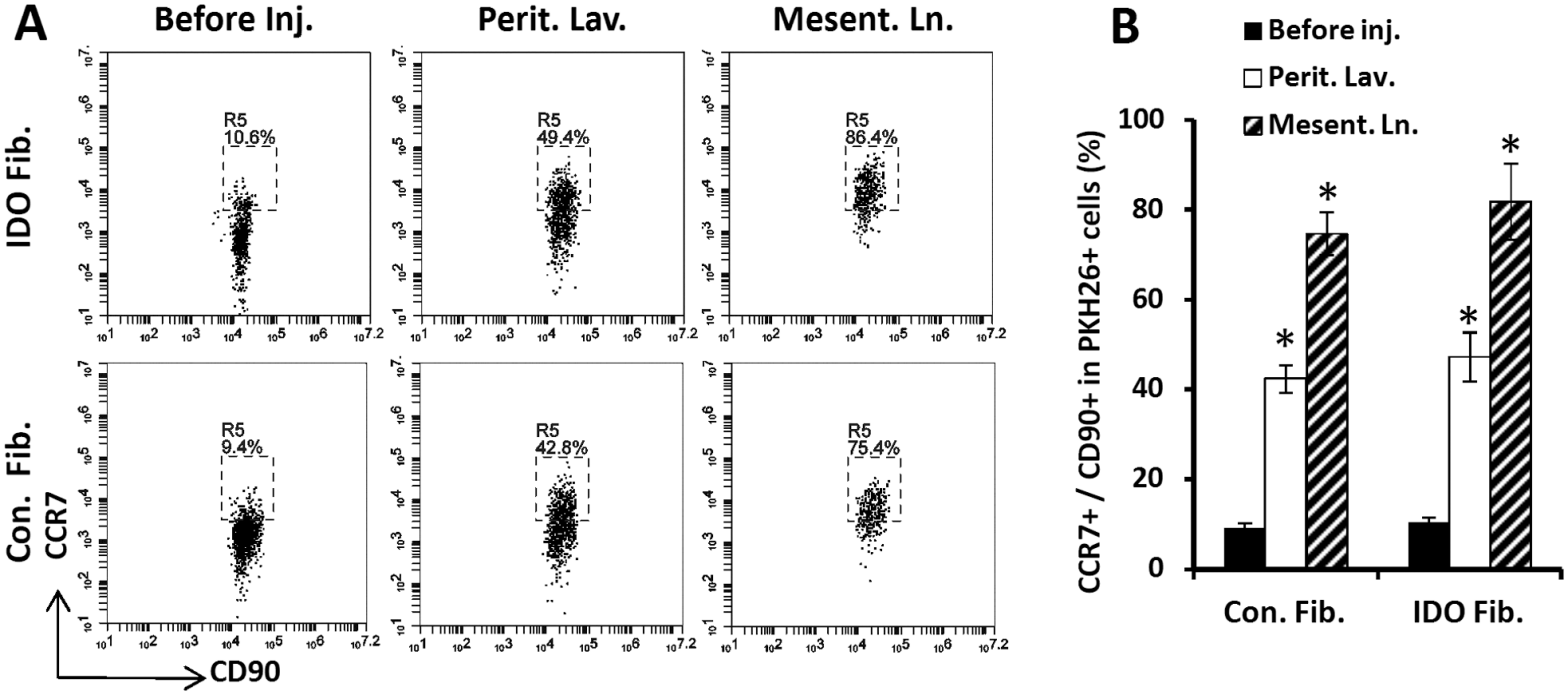
Expression of CC-chemokine receptor 7 (CCR7) by dermal fibroblasts. IDO-expressing and control mouse dermal fibroblasts were examined for expression of CCR7 before and after intraperitoneal injection. To track them, fibroblasts were labeled with PKH26 red fluorescent cell membrane labeling kit. Panel **A** shows representative flow cytometry plots showing frequency of CCR7^+^ cells gated on PKH26^+^ CD90^+^ window. Upper and bottom rows show IDO-expressing and control fibroblasts data, respectively. The plots on the left side show fibroblasts before peritoneal injection (Before inj.). The middle plots and right side plots show cells that were harvested from peritoneal cavity by lavage (Perit. Lav.) or extracted from mesenteric lymph nodes (Mesent. Ln.) two weeks post IP injection, respectively. Panel **B** show quantification of CCR7 expression on IDO-expressing and control fibroblasts before and after IP injection in peritoneal cavity and mesentric lymph nodes. (*) denotes statistically significant difference in CCR7 expression on IDO and control fibroblasts following IP injection mice compared to the before injection level (P<0.0001, *n* = 5).

Chemokine system is the essential regulator of cell trafficking in the body. In particular, chemokine receptor 7 (CCR7) or CD197 controls cell migration to secondary lymphoid organs. As such, we examined the presence of CCR7 on fibroblasts to investigate how IP injected fibroblasts homed to regional lymph nodes. As shown in Fig 6, we found that following IP injection, CCR7 was highly upregulated on both IDO-expressing (47.3% ± 8.5% vs. 10.3% ± 1.2%, P< 0.0001) and control fibroblasts (42.4% ± 3.1% vs. 9.2 ± 1.0%, p<0.0001). Moreover, majority of fibroblasts that homed to regional lymph nodes (i.e. mesenteric lymph nodes) expressed CCR7 on their surface (81.9% ± 8.5% and 74.7% ± 4.9% in IDO and control fibroblasts, respectively). These data suggest that upregulation of CCR7 is a possible explanation for fibroblast homing to regional lymph nodes following IP injection.

## Discussion

In this study we showed that an intraperitoneal IDO cell-based therapy, via a single IP injection, effectively induced and sustained long-term normoglycemia in recently yet severely diabetic NOD mice. This is in line with previous studies showing the possibility of reversal of established diabetes at early stages with no exogenous replacement of β cells precursors [23,24]. This important finding suggests that controlling autoimmunity at a crucial time during the process of diabetogenesis can restore β cell function.

The novel feature of our cell therapy model is the application of fibroblasts for treatment of autoimmune diabetes. A wide variety of cells have been used for T1D cell therapy including various types of stem cells [4,5,25], dendritic cells [26], splenocytes [27], Sertoli cells [17], or combinations of cells [28]. However, these approaches may face considerable limitations for clinical application. For instance, stem cells carry the risk of neoplasms, and dendritic and sertoli cells are hard to isolate and maintain in culture. In contrast, fibroblasts are abundant and resilient mesenchymal cells, which are very easy to isolate and maintain in culture in large quantities. Importantly, fibroblasts can function as antigen presenting cells in different tissues [29–34]. It has been shown that mesenchymal stem cells, besides their regenerative potential, are also potent modulators of the immune system. A study by Ben Nasr et al. showed the role of mesenchymal stem cells (MSCs) in the modulation of inflammatory/immune responses in delaying islet allograft rejection through multiple mechanisms [35]. Fibroblasts are very similar to mesenchymal stem cells from many aspects, can modulate the immune system [36–39], and even suppress inflammatory responses [40]. Further, as presented in Fig 4A, we found that fibroblasts can express important co-inhibitory molecules programmed cell death ligand 1 and 2 (PD-L1, PD-L1). PD-Ls are transmembrane proteins that play a major role in suppressing the immune responses in cancer [41], allotransplantation [42], and autoimmune disease [43]. PD-L1 and PD-L2 are also present on activated tolerogenic dendritic cells and play an important role in tolerance induction [43]. Fiorina et al. showed that mobilized autologous hematopoietic stem cells expressed high levels of PD-L1 and promoted engraftment of islet cell transplantation via a PD-L1-dependent mechanism [44]. Further, Guleria et al. investigated the cellular mechanisms underpinning the role of PD-1 /PD-L1 pathway in autoimmune diabetes. They found that PDL1-mediated regulation is critical for autoimmune diabetes development by limiting the expansion of CD4^+^ and CD8^+^ autoreactive T cells [45]. This group also studied the role of ICOS-B7h pathway in autoimmune responses in NOD mice [46], which is another potential pathway that can be targeted by cell-based T1D immunotherapies.

Besides having immune regulatory function, fibroblasts can produce and secret a variety of growth factors and other trophic molecules that can promote β cell viability and regeneration [47]. Overall, these characteristics make fibroblasts a very suitable choice for T1D cell therapy.

To boost tolerogenic activity of fibroblasts, we equipped these cells with IDO. IDO has been well described as a potent regulator of the immune system with indispensable role in induction and maintenance of auto- and allo-tolerance [48–51]. Increasing evidence confirms an immunoregulatory capacity for IDO in creating immune tolerance and suppression of allo/auto immune responses (reviewed in [52]). IDO enzymatic activity was instrumental for the success of our model. As evidenced in Fig 1B, neither non-IDO expressing fibroblasts nor those that were blocked by an IDO inhibitor could reveres autoimmune diabetes. Our findings as presented in Fig 3, confirms the tolerogenic function of IDO-expressing fibroblasts as the frequency of CD4^+^ CD25^+^ FoxP3^+^ Tregs significantly increased in spleen and pancreatic lymph nodes after IDO cell therapy. The capacity of IDO-expressing fibroblast in boosting Tregs was also confirmed in an *in vitro* setting as shown in Fig 5, which is in line with our previous report [20]. Aligned with the anti-inflammatory effects of fibroblasts and IDO, frequencies of two highly inflammatory T cell subsets (i.e. Th_17_ and CD8^+^ autoreactive T cells) were significantly decreased following IDO cell therapy. Indeed, CD8^+^ autoreactive cells were almost completely cleared from pancreatic lymph nodes and spleen. These findings provide a strong support for the tolerogenic function of IDO-expressing fibroblasts in autoimmune diabetes setting.

A very intriguing question in our model was to find out the path and fate of fibroblasts after IP injection. As presented in Fig 4B–4E, peritoneal cavity and its draining lymphatic system were the main destination for IP injected fibroblasts. Interestingly, both IDO-expressing and control fibroblasts made their way into mesenteric and retro-peritoneal lymph nodes but IDO expressing fibroblasts survived longer than control fibroblasts within lymphoid system. Additionally, we found that CCR7 was highly expressed on fibroblasts after IP injection. It has been shown that dendritic cells, following their activation in peripheral tissues, upregulate expression of CCR7 which enables them to enter terminal lymphatics (that express CCL21) and migrate to lymph nodes via afferent lymphatics [53–54]. As such, it is possible that upregulation of CCR7 on fibroblasts following IP injection attracts them towards peritoneal lymphatics and results in their homing to regional lymph nodes. The observation that majority of both IDO-expressing and control fibroblasts expressed PD-L1 in regional lymph nodes is in support of the idea that they maintain their suppressive capability after IP injection and migration. However, as Tregs were only increased in IDO group, it can be concluded that the combination of tolerogenic co-inhibitory phenotype and IDO is required for a robust immune tolerance induction. The fact that no labeled fibroblasts were found in blood stream, spleen and distant lymphatic system suggest that IP injected fibroblast migratory capacity is limited only to regional lymphatic system. This finding is an important piece of evidence in support of long-term safety of our cell therapy model as it shows injected fibroblast would not invade vital organs. In fact, we did not find any neoplastic lesion or tumor in our fibroblast treated mice over the course of their treatment.

**Fig 7:**
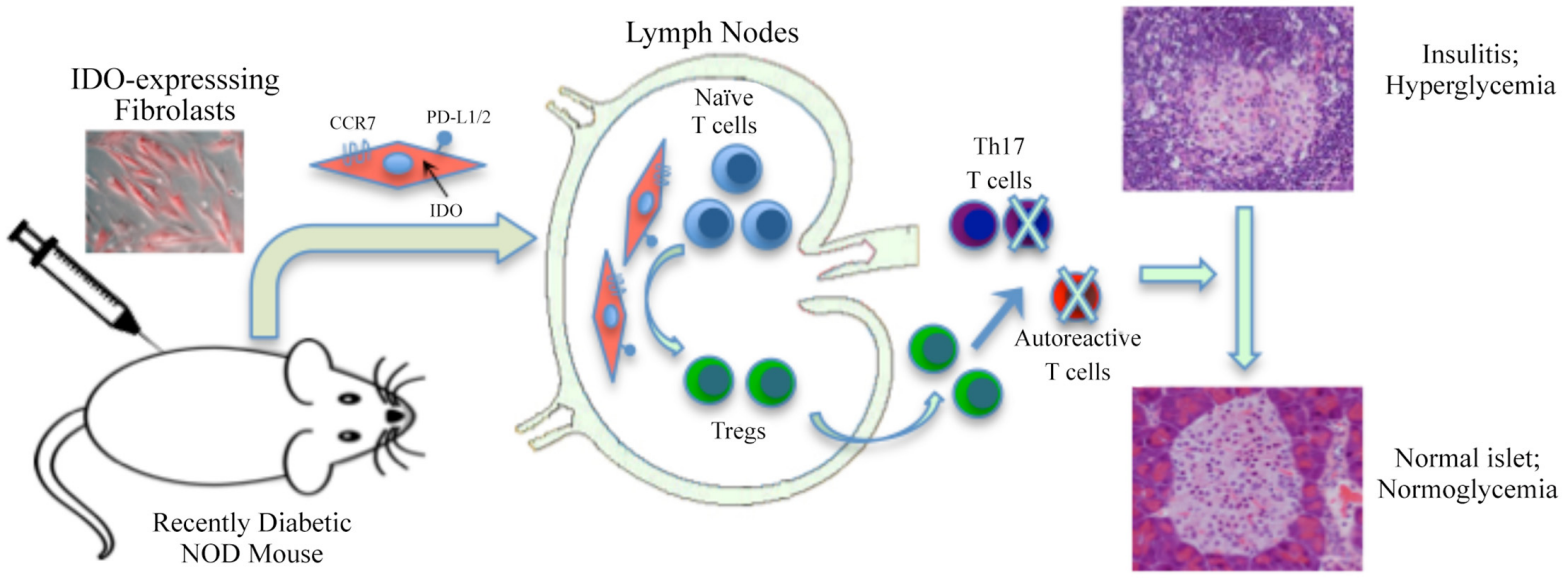
Schematic illustration of the possible mechanism for IDO fibroblast therapy model. IDO-expressing fibroblasts are injected to recently diabetic mice, intraperitoneally. A group of these fibroblasts express CCR7 and migrate to regional lymph nodes. These fibroblasts get in touch with naive T cells there and via IDO and PD-L1/2 medicated mechanisms convert them into regulatory T cells. These Tregs will then reach pancreas through blood and lymphatic streams and suppress autoimmune and inflammatory effectors immune cells. As a result, insulitis level diminishes and consequently diabetes is controlled.

The possible mechanistic scenario we propose based on these findings is illustrated in Fig 7. In this model, following IP injection, some fibroblasts express CCR7, migrate to regional lymph nodes, meet T cells there and via IDO and PD-L1/2 medicated mechanisms convert them into regulatory T cells. These Tregs then enter blood and lymphatic streams and suppress autoimmune and inflammatory effectors immune cells, ultimately resulting in diabetes remission. There are, however, other mechanistic possibilities that require more investigation. For instance, there might be a tolerogenic interaction between injected fibroblasts and host immature antigen presenting cells (APCs) resulting in polarization of these APCs toward a tolerogenic phenotype. Such a fibroblast-APC interactive model can explain the long-term maintenance of diabetes-free status in NOD mice despite clearance of IDO-expressing fibroblasts after a few weeks.

In summary, in this study we found that a single-dose injection of IDO-expressing fibroblasts is sufficient for inducing and maintaining the remission of diabetes in NOD mice. Different mechanistic aspects of this promising finding need to clarified to refine and advance it into a translational model for treatment of clinical T1D in future.

## Supporting Information

S1 Fig Tracking intraperitoneal-injected IDO–expressing and control fibroblasts. Fibroblasts were labeled using PKH26 and injected intraperitoneally. The presence of labeled fibroblasts were then checked in cervical lymph nodes, spleen, blood circulation, and pancreas after 2,4,6 and 8 weeks post-injection
(PDF)
