# Supplementary material for: Fibroblast Cell-Based Therapy for Experimental Autoimmune Diabetes
Source: PLoS One. 2016 Jan 14;11(1):e0146970. doi: 10.1371/journal.pone.0146970 (PMC4713151; doi:10.1371/journal.pone.0146970)
Supplement: S1 Fig — Fibroblasts were labeled using PKH26 and injected intraperitoneally. The presence of labeled fibroblasts were then checked in cervical lymph nodes, spleen, blood circulation, and pancreas after 2,4,6 and 8 weeks post-injection (PDF) [file pone.0146970.s001.pdf]

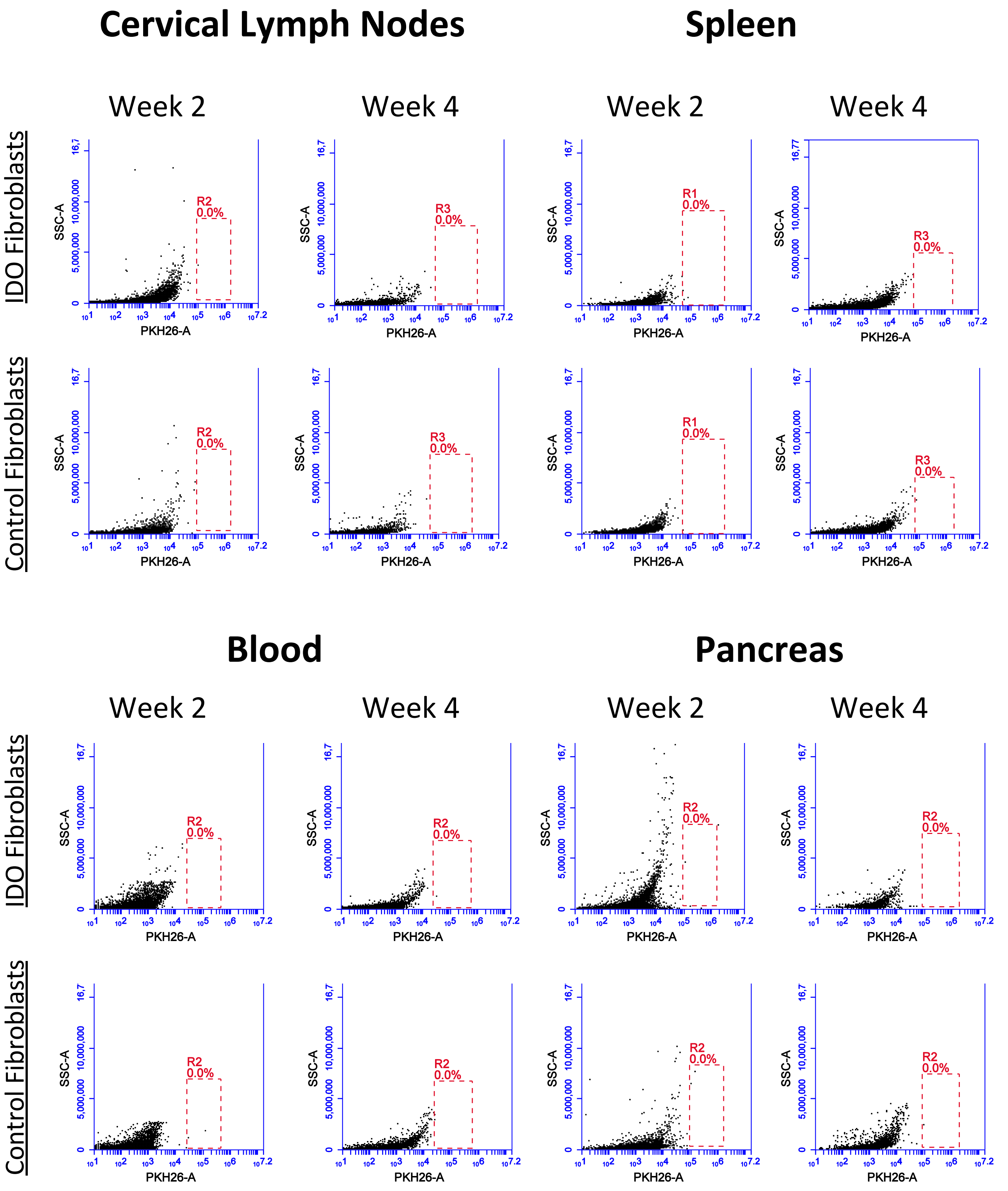
Cervical Lymph Nodes
Spleen
Blood
Pancreas
Week 2
Week 4
IDO Fibroblasts
Control Fibroblasts
SSC-A
PKH26-A
R1
R2
R3
0.0%
